# Representing Black, Asian and Minority Ethnic Skin in Dermatology Education Amidst the COVID-19 Pandemic: An Evaluation of an E-learning Resource

**DOI:** 10.7759/cureus.20738

**Published:** 2021-12-27

**Authors:** Rucira Ooi, Sheryl Li Xin Lim, Setthasorn Zhi Yang Ooi, Alistair Bennett

**Affiliations:** 1 Medicine, Cardiff University, Cardiff, GBR; 2 General Medicine, Salford Royal Hospital, Manchester, GBR; 3 General Practice, Health Education Improvement Wales, Wales, GBR

**Keywords:** ethnic minority, asian, black, representation, e-learning, dermatology, covid-19

## Abstract

Background

Recently published literature has shed light on the lack of representation and exposure to Black, Asian and Minority Ethnic (BAME) skin types in dermatology education and training. This may lead to diagnostic uncertainty and impact the overall quality of care delivered. Thus, this paper discusses the design and development of an e-learning resource as an innovative solution to address this educational need during the pandemic.

Methods

A focus group was conducted to assess the learning needs of trainees on cutaneous manifestations of BAME patients. An e-learning resource was created using instructional design, educational and multimedia principles such as the ADDIE (Analysis, Design, Development, Implementation, Evaluation) model and Gagne’s nine steps. The e-learning was disseminated to trainees across a one-month period. Feedback on the content and relevance of the e-learning was collected on the completion of the module.

Results

Overall, 84% (n=42) of trainees reported that the module improved their confidence and knowledge acquisition of common skin conditions in BAME skin types (p<0.0001 when compared to pre-course confidence). 94% (n=47) of trainees agreed or strongly agreed that the e-learning met their learning needs as an educational tool. Of 50 trainees, the results show that trainees agreed or strongly agreed that the resource was accessible (94%, n=47), reusable (94%, n=47) and promotes a sustainable way of teaching in dermatology (96%, n=48).

Conclusion

A well-structured virtual learning module can be an effective tool to deliver teaching remotely whilst complying with public health measures to prevent the spread of COVID-19. This e-learning also highlights the need for further BAME representation in published dermatological resources.

## Introduction

The COVID-19 pandemic has caused a remarkable strain on the resources available on the National Health Service (NHS). Some of the common clinical challenges faced by over 50,000 junior doctors during the peak of the pandemic include increased working hours and lower levels of support from peers and supervisors [1]. This increased workload has caused major disruption to teaching, training, and delivery of educational activities which have negatively impacted junior doctors’ training needs for career progression. While this upheaval has posed unprecedented challenges to our services, the pandemic has catalysed the rapid adoption of telemedicine and revolutionised the way we deliver care remotely [2,3,4]. There is, however, also a need to continue providing high-quality training and education to junior doctors whilst complying with public health measures. Fewer opportunities for educational activities impose a need for us to transform and innovate current resources into accessible education initiatives.

COVID-19 has altered training programmes by reducing the amount of hands-on time physicians could spend with patients. Consequently, junior doctors currently training in primary care may lack clinical experience in recognising and treating common dermatological conditions, despite having approximately one in four patients presenting at their General Practice (GP) with skin problems each year [5]. The authors also report a lack of exposure to a diverse patient population due to the geographical location of their workplace, a General Practice surgery in South West Wales, and a district general hospital in North West England, where the population predominantly consists of 98.1% and 96.7% of White British residents respectively [6,7]. This adds an extra layer of barrier in allowing junior doctors working in similar areas to appreciate lesions that may present differently in patients of a darker skin tone. Additionally, clinical supervisors who have only ever worked in these geographical areas will also have limited experience. Workplace-based assessments too are unable to provide these learning opportunities if there is minimal exposure to various patient groups.

The lack of representation and exposure to darker skin types in education and training may suggest that there is an element of diagnostic uncertainty and inaccuracy which may result in a shortfall in the quality of care delivered. There are also limited published educational resources on dermatological conditions seen among Black, Asian and Ethnic Minority (BAME) patients. Furthermore, when considering the current state of postgraduate medical education, the majority of formal teaching and learning is delivered in a traditional didactic method [8]. Since identifying these knowledge gaps in dermatology, in the workforce, the authors initiated the delivery of a virtual dermatology learning environment to address these issues: (i) the lack of face-to-face clinical exposure to common dermatological skin lesions due to public health measures during the COVID-19 pandemic, (ii) the need to increase the representation of different skin tones in dermatology teaching, and (iii) the need to deliver effective, interactive and high-quality virtual teaching alike in-person teaching events. 

Recent publications in medical education suggest that virtual teaching is increasingly popular during the pandemic due to its effectiveness in delivery [9]. Nevertheless, the delivery of virtual learning presents its own challenges too, which commonly include reduced participant engagement and technical difficulties. Therefore, the authors produced a user-friendly e-learning module, accessible on all web browsers, which incorporates the use of interactive tools and relevant content to ensure optimal engagement. Its development was based on a structured framework known as the ADDIE (Analysis, Design, Development, Implementation, Evaluation) model and its contents using Gagne’s nine steps of instruction [10,11].

The authors hypothesised that this approach would encourage trainees to support one another to ensure training and educational needs are met despite the limitations during this pandemic.

## Materials and methods

The design and development of the online learning resource were based on instructional design, educational and multimedia principles. A literature search on designing and implementing training identified that the ADDIE model was commonly used amongst educators, instructional designers and training developers [11]. Several studies have also demonstrated that the defined stages of the ADDIE model have acted as an effective guide for designers to organise teaching content and aid the implementation of teaching tools [11]. Therefore, the methods of the study will be described according to the 5 stages of the ADDIE model.

Analysis: assessing learning needs and identifying learning objectives

A focus group of 10 junior doctors was conducted to assess the learning needs of trainees on cutaneous manifestations of BAME patients before the development of e-learning. A PACT (People, Activities, Contexts and Technologies) analysis was carried out to clearly define the learning objectives for the e-learning of which users are expected to achieve upon completion of the module; the settings under which they need to perform them and the acceptable standard, before relevant learning activities and assessments are designed [12]. Discussion and responses from the PACT analysis and focus group identified several intended learning outcomes. Using Bloom’s Taxonomy and SMART (Specific, Measurable, Achievable, Realistic, Time-bound) aims, the learning outcomes were created and described in the introductory slide of the e-learning resource (Table 1) [13].

**Table 1:** The intended learning outcomes introduced at the start of the e-learning using Bloom’s taxonomy and SMART framework

SMART Framework	Learning Outcomes
Specific	To be able to recognise different common skin lesions in patients of non-white skin tones
	To be able to formulate and plan appropriate basic investigations and a plan of management
Measurable	To be able to assess the learners' understanding of the topic through performance of quizzes and other interactive elements
Achievable	To be able to identify and apply the key characteristics and types of common skin lesions in primary care and medical practice
	To help users build confidence in seeing patients of various ethnicities presenting with skin lesions
Realistic	To be able to recall the presentation of common skin lesions in other skin tones by participating in the e-learning’s interactive activities such as spot diagnosis and memory game
Time-bound	To be able to achieve the intended learning outcomes (ILOs) in the one-hour session of e-learning

Design and development: conceptualising and creating the e-learning module

The design process involved the production of a prototype using a storyboard to demonstrate the layout of the e-learning to the authors and to aid the development of the multimedia resource. The authors then adopted a virtual software, Xerte Online Toolkits to produce a 20-slide e-learning resource [14]. As dermatology is a field that relies highly on visual cues, this interactive virtual learning resource was created with numerous images obtained from reliable and licensed dermatological atlases [15,16]. The 20-slide e-learning resource consisted of common skin conditions that frequently present at primary care providers [17]. Images were used to display and describe its presentation in different skin types.

Furthermore, the authors utilised Gagne’s nine steps of instruction to create efficient and effective learning experiences for users, as demonstrated in Table 2.

**Table 2:** The application of Gagne’s nine steps on the content and learning activities of the e-learning resource

Gagne’s Nine Steps	Description
Gain attention (Reception)	The introduction slide highlights the importance of raising awareness on the presentation of common skin lesions in other skin tones to stimulate interest in the subject matter.
Inform learner of objectives (Expectancy)	A list of intended learning outcomes is introduced to enable the learner to work towards achieving them throughout the learning module.
Stimulate recall (Retrieval)	Two pre-course case studies challenge the learner to recall any prerequisite learning obtained during their training, enabling them to prepare for new teaching content in the subsequent slides
Present stimulus material (Selective perception)	This strategy is incorporated into the main body of the learning resource with the introduction of the ABCDEs (Asymmetry, Border, Colour, Diameter, and Expert) of examining a skin lesion followed by the definition of the Fitzpatrick scale which is used to identify different skin types and their associated risk to ultraviolet (UV) light. Further information on the history, examination, management, and appearance of seven common skin lesions are portrayed in different skin types.
Provide learner guidance (Semantic encoding) & Elicit performance (Responding)	The use of post-course case studies and memory games help students to learn the material. These activities aim to help the learner internalise new knowledge and provide an opportunity for the learner to confirm their understanding of the resource.
Provide feedback (Reinforcement)	Learners are presented with a feedback form to reflect on the content, relevance and presentation of the resource.
Assess performance (Retrieval)	Quizzes assess the performance of the learner and provide immediate feedback if an incorrect answer is chosen. Further explanation in the form of written and visual images to each answer option is provided.
Enhance retention (Generalisation)	Learners are also asked to identify three things that they have learned or found useful from the e-learning. These questions were designed to provide an opportunity for learners to recall and apply what they have learned during the resource and consequently help them internalise the information obtained. The feedback form also presents as a platform for learners to reflect on their performance of the module, with an aim to reduce the impact of cognitive dissonance.

Implementation and evaluation: application and appraisal of the e-learning module

The e-learning module was disseminated to junior doctors in both study locations through email and was given a month to complete. Upon completion of the Xerte e-learning module, users are provided with a 15-item, self-administered feedback form to evaluate the quality of the content and relevance of the e-learning resource and the impact it has on their confidence in practice (Table 3). The evaluation tool was created using the Learning Object Review Instrument (LORI) developed by the e-Learning Research and Assessment Network (eLera) and the Portal for Online Objects in Learning (POOL) [18]. The LORI is commonly used to evaluate online learning resources [18].

**Table 3:** List of questions from the 15-item feedback form presented to learners upon completion of the learning resource

Questions in the feedback form
1. What would you rate the quality of the content presented (accuracy, balanced presentation of ideas, appropriate detail)? (Likert scale)
2. How strongly would you agree that the module has reflected on the intended learning outcomes? (Likert scale)
3. How confident did you feel about identifying common skin lesions on various skin tones before the learning module in clinical practice? (Likert scale)
4. How confident did you feel about identifying common skin lesions on various skin tones after the learning module in clinical practice? (Likert scale)
5. How would you rate the organisation and presentation of questions/quizzes in the module? (Likert scale)
6. How would you rate the organisation and presentation of learning points/images in the module? (Likert scale)
7. How would you rate the sustainability of the learning module in Dermatology? (Likert scale)
8. How would you rate the accessibility of the learning module? (controls and presentation formats to accommodate disabled and mobile learners) (Likert scale)
9. How would you rate the reusability of the learning module? (ability to use in varying learning contexts and with learners from differing backgrounds) (Likert scale)
10. How would you rate the aesthetic of the learning module? (contrasting text colours, font size, text font, image quality) (5-point rating)
11. How likely would you recommend this learning module to a colleague or friend? (5-point rating)
12. How would you rate this learning module overall? (5-point rating)
13. Do you have any other feedback for the tutor? (Free text)
14. List 3 things that you have learned or gathered from this module. (Free text)
15. List 3 ways the tutor could improve on the design of this module. (Free text)

Data processing and storage

The feedback form included a five-step Likert scale, free-text questions, and ratings to improve the granularity of the data. To prevent re-entrance of data, each response was limited to a single valid email address. Throughout the period of data collection, the information received was kept in a password-protected file. All relevant information remained non-identifiable throughout this study.

Statistical analysis

Two individuals, RO and SZYO, analysed the data independently and cross-checked the results. AB reviewed and resolved any discordance in results. A combination of qualitative and quantitative analysis was used. A paired t-test was used to analyse whether the learning module had a significant impact on users' confidence in identifying common skin lesions in BAME patients. Numerical values 1 to 3 are assigned to correspond to the responses 'not confident (corresponds to 1), neutral (2), and confident (3)’ respectively. A p-value of 0.05 was set to be significant in this study. Microsoft Excel (Microsoft, Redmond, Washington, USA) was used for data analysis and graph production.

Ethics and dissemination 

Participation in the survey was completely voluntary and confidential. Responses provided were anonymous. Upon submitting the forms, participants confirmed their consent to participate in the survey and to the handling of data according to Article 6(1)(a) of the General Data Protection Regulation (GDPR). Information was held in confidence in accordance with GDPR principles. No data on any protected characteristics of the individuals were collected. Individuals were allowed the right to withdraw consent and request removal of their data from the Microsoft Form platform at any time. Access to the data was only granted to the authors of the study. Participants were allowed to express any questions, concerns, or requests regarding the use of data to the authors. Using the NHS Health Research Authority decision tool, the survey did not need NHS Research Ethics Committee review.

## Results

Ten junior doctors participated in the focus group whilst 50 participated in the survey. These trainees were from two different study locations, a General Practice in South West Wales and a district general hospital in North West England, UK. Ten of the 50 junior doctors completed a rotation in General Practice. Participants were made up of junior doctors who are currently in either the 1st or 2nd year of the Foundation Programme, and are not specialists in dermatology.

Focus group

Of the 10 in the focus group, seven (70%) expressed a further need for BAME representation in dermatological teaching (Table 4a). Five participants (50%) supported the use of e-learning educational tools as an accessible resource for junior doctors, General Practitioners and medical students (Table 4 b).

**Table 4:** Thematic analysis of free-text responses received from feedback BAME: Black, Asian and Minority Ethnic; GP: General Practice

Themes identified	Description
a) The need for BAME representation in dermatological teaching.	“I think it is definitely a good idea! There is a need for more published resources to represent skin diseases in BAME (Black, Asian, Ethnic Minority) patients.”
	“There has been a petition going around as well, to include more BAME patients in pictures and published materials.”
	“This will be a good e-learning summarising skin diseases in BAME patients, a quick revision for clinical practice.”
b) The use of e-learning as an educational tool.	“An e-learning will be a great learning resource that can be accessible to both General Practitioners, junior doctors and medical students!”
	“I agree. Make sure you include quizzes throughout to make it fun.”
	“Make the module informative and interactive.”
	“Use photos! Make sure they are of good resolution to make it look professional.”
	“You should approach the medical school once you’ve finished creating the e-learning. Hopefully, they can recommend it to more medical students by uploading it onto Learning Central for those who are on Dermatology placements.”
c) The e-learning should be implemented as a regular teaching tool.	“More of this, please! Make this a regular teaching for us junior doctors.”
	“A recurring series of this type of e-learning will be very much appreciated. Especially because of COVID, all in-person teachings are cancelled!”
	“Although this e-learning covered common skin lesions, will suggest to the tutors to continue these as an e-learning series covering other types of skin diseases in BAME.”
d) The e-learning impacted clinical practice in primary care.	“I have learned how to describe lesions seen and identify key characteristics of skin lesions commonly presented to GP’s. I have also gained confidence in the management of these common skin conditions and therefore I hope to be able to reassure my patients with this confidence.”
	“Thank you for doing this. It’s been helpful for me to go through dermatology before starting my GP rotation.”
	“This is something I've been looking for! Aids my clinic at GP! Especially when we don't have much exposure to Dermatology in medical school too, this is the perfect toolkit for junior doctors and also medical students!”
	“I loved the content and pictures used. This is definitely a fun and interactive way to learn dermatology. Helps with us at GP placements too especially we see so many patients with skin lesions.”
	“I am planning to pursue General Practice with a special interest in dermatology. Really useful for us as a refresher.”
	“Great learning resource for junior doctors especially those dealing with Dermatology patients in primary care!”
e) The e-learning addressed the lack of BAME representation in dermatology.	“The learning module covers a very important topic on the lack of representation of BAME in dermatology resources! Great job for putting this together! I use it as a quick reference too occasionally.”
	“The e-learning identifies a gap in our teaching programmes for postgraduate junior doctors. Will be useful to share it on a national platform.”
	“Highlights a gap in our knowledge, great learning resource! I particularly liked that for the quizzes, if you got the answers wrong, you were allowed to attempt them again. The tutor also provided explanations to the wrong answers.”
	“I'm more confident in identifying/diagnosing BAME skin conditions. The memory game was fun and consolidated my learning!”
	“I am able to understand the implications of misdiagnosis of common skin diseases in BAME people.”
	“Recognition of common skin lesions and its management, acknowledgement of the lack of education and training in recognising lesions in BAME skin.”

Feedback form

Of 50 junior doctors who engaged with the e-learning, 100% (n=50) completed the learning module and filled in the feedback form.

E-learning as an Educational Tool

**Figure 1:**
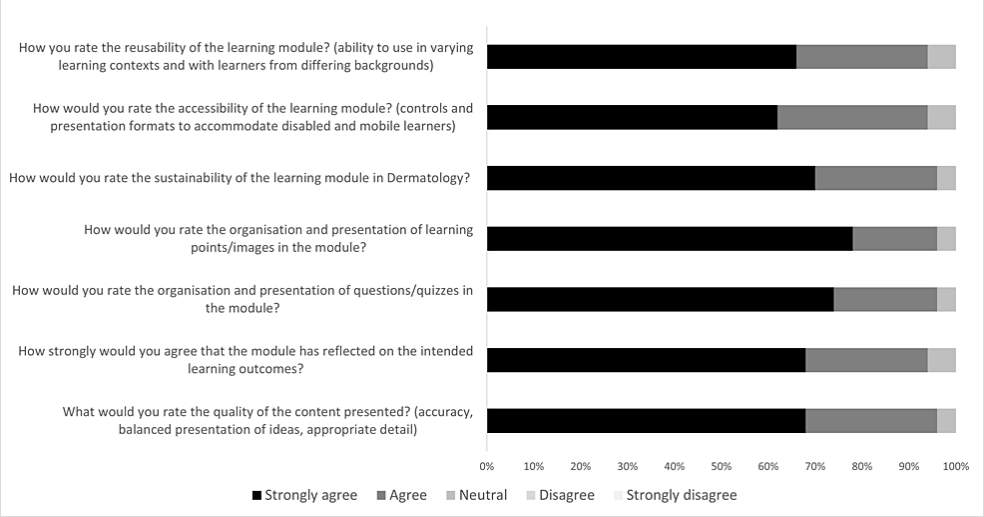
Summary of feedback responses

94% (n=47) of trainees agreed or strongly agreed that the learning module reflected on the intended learning outcomes. Trainees also agreed or strongly agreed that the e-learning was easily accessible (94%, n=47), that its format and approach is replicable in other specialties (94%, n=47) and that it promotes a sustainable way of teaching in dermatology (96%, n=48). Figure 1 demonstrates a 100% stacked bar chart that shows the percentage of responses received from the feedback.

Qualitative feedback received from 10 (20%) users suggested the implementation of e-learning as a regular teaching tool for junior doctors (Table 4c).

Impact on Clinical Practice

**Figure 2:**
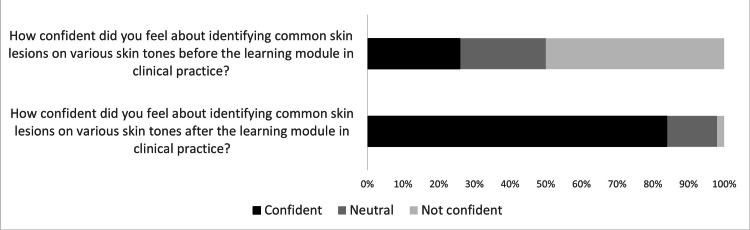
Pre-course versus post-course confidence of participants

84% (n=42) of trainees reported that the module improved their knowledge acquisition of common skin conditions in BAME skin types, hence, improving their confidence in clinical practice. The paired t-test showed that the e-learning module had a significant impact on users' confidence pre-course (mean = 1.76, standard deviation = 0.85) versus post-course (mean = 2.82, standard deviation = 0.44) (p<0.0001). Figure 2 demonstrates the percentage of responses received from the feedback.

Ten (20%) users found that the learning module helped them identify and manage dermatological conditions in other skin types, and improved their clinical practice in primary care (Table 4d).

Addressing the Lack of BAME Representation in Dermatology

Twenty-five (50%) of junior doctors agreed that the contents of the e-learning were relevant and addressed the need for more BAME representation in dermatology (Table 4e).

## Discussion

This is the first study to evaluate the use of an e-learning resource in teaching dermatology whilst increasing awareness of BAME skin representation. Feedback from our educational tool demonstrated a significant increase in the participants' confidence in diagnosing and managing dermatological conditions.

Lack of representation of BAME patients in published dermatological resources

COVID‐19 has impacted people of colour disproportionately. In the UK, the death rate for people of Afro-Caribbean descent was 3.5 times higher than for White British people whilst in the United States (US), African Americans represented 33% of COVID-19 hospitalisations, despite only making up 18% of the total population studied [19]. Recent studies have also shown evidence that COVID‐19 impacts several organ systems, including the skin [4,20]. In some cases of COVID-19, a rash may occur as a manifestation of the disease without viral symptoms such as a continuous cough, fever, or loss of smell [21]. Despite this, there is still a lack of diversity in images showing coronavirus-related skin manifestations [21]. A systematic review by Lester et al included an analysis of 130 published clinical photos of COVID-19 cutaneous manifestations and unexpectedly found not even one photo featured patients with Fitzpatrick skin types V or VI [22]. The knowledge of cutaneous manifestations of COVID‐19 and the ability to identify them in patients of all skin types is important for dermatologists and other healthcare providers who may be evaluating otherwise asymptomatic patients to ensure safe, reliable care for all patients.

Several studies have also shown evidence of the lack of appropriate diagnosis in darker skin tones leading to poorer outcomes [23]. Studies have also demonstrated that melanomas such as lentigo maligna are easily missed in African patients leading to a very late diagnosis [24]. Some conditions such as Lyme disease are underdiagnosed due to its unusual presentation in other skin types [25]. The recognition of life-threatening conditions such as cyanosis and hypoxia could also be missed if the emphasis in published resources is on ‘blanching’. The use of appropriate language to describe the presentation of dermatological signs in non-White patients is very important as current terminology assumes a ‘White norm’ such as rubra and pallor. These diseases, which manifest differently in different skin tones, were included in our resource to bridge the gap in published dermatological resources.

Effectiveness of virtual learning

The majority of trainees agreed or strongly agreed that the e-learning met their learning needs as an educational tool. This is in keeping with the increased use of webinars and e-learning during the pandemic, due to its practicality and cost-effectiveness [3]. The authors can only envision that this will continue well into the post-COVID era. Besides, Continuing Medical Education (CME) activities are increasingly shifting to virtual platforms. It may be that future CME activities embrace a hybrid approach of blending digital learning with face-to-face sessions. Technological interventions such as Microsoft Teams^TM^ are also increasingly utilized during the pandemic to facilitate meetings with colleagues, telemedicine consultations with patients and online interviews [26].

Remote consultations during the pandemic required clinicians to formulate their differential diagnoses based on digital images via telemedicine [3]. The ability to accurately recognise and classify a range of skin signs and symptoms on different skin types is an essential skill and is traditionally acquired through extended clinical experience and exposure. However, this e-learning has proven its effectiveness in increasing clinicians’ confidence in making a spot diagnosis on inspection of a digital image during service provision via telemedicine.

Sustainability in dermatology

This e-learning aimed to bridge the knowledge gap amongst junior doctors and to raise the awareness surrounding the lack of representation of BAME patients in dermatology teaching, published textbooks, and journals. In July 2020, an article by the British Medical Journal (BMJ) highlighted the success of a 2nd-year medical student in publishing a book called ‘Mind the Gap’ which subsequently led to a petition signed by nearly 200,000 people to call for proper training of doctors in identifying rashes present in the BAME population [27]. BAME patients may not present in the same way as White patients and are not as clinically obvious; hence, representation in educational resources can improve diagnostic accuracy.

The COVID-19 pandemic has been the “magnifying glass” that society needed to realise the ongoing racial disparities in healthcare. Likewise, in the wake of the Black Lives Matter protests, healthcare professionals have been called upon to diversify teaching at medical schools and in clinical textbooks [28]. Additionally, as NHS organisations are accountable for continuously improving the quality of their services by safeguarding a high standard of care, clinical governance assures that the wide differences in the quality of care throughout different NHS trusts in the UK are addressed. Hence, junior doctors also play a role in practising clinical governance through developing teaching skills as part of their professional development. Besides, the Good Medical Practice guidance by the General Medical Council (GMC) suggests that all doctors are required to develop and maintain professional performance by ensuring competency in all aspects of work, encompassing teaching [29]. Therefore, junior doctors should be prepared to contribute to teaching and training fellow doctors and students. This e-learning provides junior doctors with an innovative solution to continue teaching activities despite current public health measures.

Limitations

The biased-based approach of the study, which mainly included participants who are practising in areas where the population are predominantly White British, means that the views of the focus group and survey respondents may not represent the views nor have similar pre-course insights as that of the junior doctors across the country. However, this approach was also the main strength of the study; it allowed better discernment for the junior doctors who are affected due to the location of their practice. Hence, the results of the study provide an accurate representation of the challenges faced by the doctors practising in similar areas, thus, allowing a more accurate assessment of the effectiveness of the tool. Besides, the study only looked at levels 1 and 2 of Kirkpatrick’s hierarchy of evaluation [30]. Therefore, further work requires evaluation of the e-learning resource in eliciting behavioural change and improving patient care.

The authors also acknowledge that there are potential drawbacks of representing darker skin with pathology. The risk of racialising and causing racialised bodily stigmatisation are to be considered when representing BAME skin in dermatology education. Moving forward, representation of BAME skin in medical education is encouraged to avoid prototypical cases where a condition is strongly associated with a certain ethnic group. There is also an assumption that ‘common skin conditions’ present the same way in non-BAME groups, however, this is not true. The ‘classic’ or ‘textbook’ presentation of rashes are not common in any skin tone. 

Lastly, the authors acknowledge the small sample size of the present study. Future studies with a larger sample size would be beneficial in increasing the validity and generalisability of the reported usefulness of the resource. 

## Conclusions

Dermatological health disparities have become increasingly apparent in recent years. This study has demonstrated the beneficial use of an e-learning resource in improving the knowledge of junior doctors on recognising and managing BAME skin conditions. The authors aim to disseminate the e-learning resource to all junior trainees via local postgraduate programme coordinators and to medical students via dermatology societies and medical schools. Teaching on this topic should start earlier in medical school, not only during dermatological placements. We advocate for more teaching on BAME skin conditions through e-learning modules as it has shown great promise as an effective tool to deliver teaching in dermatology remotely.
